# Leading schools during the COVID-19 school closures in Estonia and Finland

**DOI:** 10.1177/14749041221138989

**Published:** 2022-12-02

**Authors:** Raisa Ahtiainen, Eve Eisenschmidt, Lauri Heikonen, Merilyn Meristo

**Affiliations:** University of Helsinki, Finland; Tallinn University, Estonia; University of Helsinki, Finland; Tallinn University, Estonia

**Keywords:** School leaders, leadership practices, crisis leadership, COVID-19, Estonia, Finland

## Abstract

We examined practices and experiences of leadership during the COVID-19 school closures in 2020 by analyzing Estonian (*N* = 219) and Finnish (*N* = 775) school leaders’ responses to open-ended questions in an electronic survey. In the analysis we used five categories drawn from the data: Making decisions, Re-organizing schoolwork, Communication, Giving support, and Self. The findings show leaders stressing especially the themes of within school communication and giving support both at emotional-level and in the use of technology in teaching. The findings signal a leadership approach composed of care and empathy. Moreover, within the category Self, the leader’s ability to lead themselves and manage stress were extensively brought out. We provide four messages reflecting leadership during times of both turbulence and tranquility. For example, a leader’s knowledge about pedagogy prepares the ground for meaningful reorganization of teaching and learning. Also, attention should be paid to locally established structures for collaboration across schools as they support leaders’ work.

## Introduction

During the early months of 2020, countries around Europe reacted with a range of measures to suppress the spread of COVID-19 (Kelly et al., 2021; Lindblad et al., 2021; Marques da Silva, 2021). Most countries closed schools and gave instructions for alternative ways of education detaching learning and teaching—and other daily interaction—from their usual environment, formed of physical space of school premises (Lindblad et al., 2021). Although the decisions concerning school closures were made mostly at the ministerial or government level, in many countries the decision-making involving implementation of remote learning occurred at the local level (Campbell, 2020; Hargreaves and Fullan, 2020; Kelly et al., 2021; Marques da Silva, 2021) and that applied also to Estonia and Finland shifting to remote learning in March.

In the European context, the shift to remote learning can be considered as an acceleration and consolidation of the implementation and use of a variety of technologies in schools (Cone et al., 2022; Kelly et al., 2021). Nevertheless, the ethos of digitalization of education had already before COVID-19 formed a backdrop for many national education systems—at least at the strategic level (Cone et al., 2021). Therefore, in many places (e.g. in Scandinavia) teachers and students were familiar with the use of technology in learning and instruction, and the sudden closure of schools only pushed forward the reforms around the digitalization of schoolwork (Cone et al., 2022). That is, the organization of virtual lessons depended on how well teachers were prepared to use technology, how quickly schools responded to the need to develop teachers’ digital competence, and what was the overall availability of internet access and digital devices (Kelly et al., 2021).

From the perspective of school leaders’ work, the era of the COVID-19 pandemic has brought up challenges culminating in experiences of uncertainty and ambiguity (Marshall et al., 2020). Leaders’ work has taken place in school communities going through a spectrum of emotions ranging from shock to inspiration regarding discoveries of novel solutions to problems not encountered before (Flores and Swennen, 2020). To support a sense of security for teachers, other staff (e.g. teaching assistants), students, and guardians, some leadership practices have proved to be worthwhile. Running the school in disruptive times requires more than routine problem solving or occasional firefighting. The leaders are expected to be capable of immediate and decisive action (Smith and Riley, 2012). Further, the ability to gather information, adapt it to decision-making, and to demonstrate concerns and communicate clearly are crucial (Smith and Riley, 2012).

As the earlier studies published here have noted, national education systems differ regarding their experiences on crisis or unexpected events affecting the functionality of the society (e.g. Cone et al., 2022; Kelly et al., 2021; Lindblad et al., 2021; Marques da Silva, 2021). A large part of the literature from the pre-COVID-19 era discusses factors (e.g. natural catastrophes) other than global pandemics as the cause of exceptional educational arrangements (Huber and Helm, 2020). To prepare ourselves for the unexpected changes in the future and to develop the field of education, empirical studies are needed to learn from the experiences of actors working at all levels within education systems (Beauchamp et al., 2021; Huber and Helm, 2020). This study explores practices and viewpoints regarding leadership in comprehensive schools during the COVID-19 school closures in 2020 in Estonia and Finland. Our paper contributes to school level knowledge on leadership by reporting on findings of leaders’ perceptions of the leadership and their experiences as leaders.

### Leading in and through turbulent times

Leaders having a broad understanding of change and leadership are more likely to be successful in navigating their school communities through turbulent times (Gurr and Drysdale, 2020). Leadership qualities such as the ability to synthesize and communicate information, capitalize on new opportunities, empathize, and respect diverse perspectives and personal characteristics are highly relevant in critical situations (Smith and Riley, 2012). In addition, contextual knowledge enables leaders to overcome the challenges and find ways to alter or go around the situation (Drysdale and Gurr, 2017). It is essential for a leader to understand and analyze the known and unknown aspects of the context to see what is important (Leithwood et al., 2020). Leaders are also expected to have a vision, set goals, and manage everyday challenges (Eisenschmidt et al., 2021), and to provide support for others and have a capacity to lead.

#### Providing direction

The COVID-19 pandemic led to challenges characterized by uncertainty and complexity, to which we cannot automatically apply the currently known methods (Dunn, 2020). In a school, the leader sets the direction for change and development of practices (Leithwood et al., 2020). Decision-making for the direction takes into consideration the evidence and knowledge (Gurr and Drysdale, 2020), and unpredictable circumstances require individual-level flexibility and ability to adapt to new situations (Beauchamp et al., 2021). As this process can entail unintended consequences, it requires readiness to reassess the direction and make modifications in plans and instruct others accordingly (Gurr and Drysdale, 2020; Marshall et al., 2020). That means a leadership approach that is responsive, has a clear idea of the direction, but also flexibility and adaptation to unexpected changes with an understanding that the direction may need reconsideration (Berger and Johnston, 2015; Dunn, 2020; Gurr and Drysdale, 2020).

In uncertain times, communication becomes a central means for sense-making (Gurr and Drysdale, 2020), giving direction, and creating optimism and certainty (Smith and Riley, 2012). During the school closures the meaning and aim of communication has expanded beyond its instrumental purpose (Beauchamp et al. 2021). Timely communication along with providing information and instructions have become means for expressing support and comfort (Wang 2021). Consequently, communication requires a leader’s increasing engagement and interaction in a two-way process with teachers, students, guardians, and others involved (Drysdale and Gurr, 2017). Communication can also become a focal means for building a virtual image of the school as a community and a channel for teachers and other staff to support each other (Beauchamp et al., 2021).

#### Collective leadership practices

Conflicting information concerning the COVID-19 and the methods and means of protection have added to the complexity of the situation in which school leaders work (Beauchamp et al., 2021; Marshall et al., 2020). Practices relying on interaction, collaboration and distribution of leadership tasks have been given a lot of weight in literature considering prerequisites for successful leadership during the pandemic (Beauchamp et al., 2021; Harris and Jones, 2020; Marshall et al., 2020). The concept of distributed leadership is a better-known approach for describing a model in which leadership is dispersed among many in a school community (Harris, 2008; Spillane, 2005). It has a positive influence on school and student learning outcome improvement (Heck and Hallinger, 2010), but also to the well-being of those responsible for leading the school (Hopkins, 2009). This form of working involves distribution of responsibilities and tasks and can occur in various ways depending on the school context (Harris, 2008; Lahtero et al., 2019).

Collective responsibilities and practices can foster a collaborative culture and encourage teachers to participate in school-based decision making, taking initiative (Katzenmeyer and Moller, 2009) and engage them in professional learning (Eisenschmidt et al., 2021). These features of leadership and ways of working have been found to be a source of strength among leaders in encountering unpredictable changes (Beauchamp et al., 2021). They enable the use of the expertise and knowledge of many and support leaders in their decision-making (Marshall et al., 2020). These qualities of a work community make schools more creative and responsive in dealing with a crisis (Campbell, 2020; Harris and Jones, 2020). Moreover, crises can provide schools with the potential to develop, yet this involves giving up old ways of working (McLeod and Dulsky, 2021).

#### Giving support

A sudden transition to a new way of schooling changed the nature of leaders’ work and called for more emphasis on supporting the school community (Pollock, 2020). It has been crucial to maintain a feeling of a community and nourish a positive sense of purpose and mutual support in the school. Drysdale and Gurr (2017) discuss a moral compass that covers empathy, a balance between collective and individual care, and giving a central position to people. High moral values and ethical sensitivity are paramount in challenging school contexts (Hanhimäki and Tirri 2008). It is important to look after the members of the school community and prioritize the wellbeing and coping of the people working there when guiding them through an unexpected situation (Beauchamp et al., 2021; Thornton, 2021).

Empathy as the ability to understand the perspectives of others (Combs et al., 2018) is one of the leader’s characteristics for enhancing the creation of a supportive and safe environment (Berger and Johnston, 2015). A school community composed of collaboration and support nurtures teachers’ self-esteem and self-confidence and encourages them to take responsibility for managing challenging situations (Kohm and Nance, 2013). Moreover, leaders can foster teachers’ creativity and readiness for risk-taking by guiding the implementation of new teaching methods and practices (Yukl, 2012). In a trusting atmosphere, teachers can feel safe and are more likely to ask for help and recognize other’s problems. Trust is a pre-condition for collaboration in an organization, and its development occurs through the leader’s proactive and effective conflict management (Youngs and King, 2002). The way a leader behaves builds up trust (Kruse and Louis, 2009), and the meanings and interpretations given to these actions and behaviors can lead to the creation of the feeling of safety.

#### Managing self

Leithwood (2012) has introduced the concept of “personal leadership resources” covering cognitive resources such as problem-solving expertise and systems thinking, but also social resources referring to ability to perceive and manage emotions, and act in emotionally appropriate ways. It also includes psychological resources like optimism, self-efficacy, resilience, and proactivity (Leithwood, 2012). For example, resilient leaders being capable of coping with the crisis (Beltman et al., 2011) demonstrate continual support for the members of the organization and spur on a strong commitment to the mode of action (Southwick et al., 2017). Later Leithwood et al. (2020) have concluded that these personal resources may provide a framework for explaining a high proportion of variation in the practices enacted by school leaders. In stressful events, psychological resources become even more important. The leaders who are self-aware and able to keep disruptive emotions and impulses under control, are more likely to adapt to new challenges and think positively (Nokelainen and Tirri, 2007).

Leaders are increasingly managing emotional responses to the crisis including anxiety, frustration, loss, and anger that can become emotionally exhausting (Mahfouz, 2020). Consequently, self-care must be a priority for those leading in education at all levels (Harris and Jones, 2020). One may argue that leaders should learn to lead themselves before they are able to lead others successfully. Leading a school through COVID-19 and post-COVID-19 times will require school leaders who put their own health and wellbeing first as leaders’ personal beliefs about how to best manage their own stress seem to be aligned with their orientation to work relentlessly in supporting others (Reid, 2021).

#### Present study

In this article we report on a study the aim of which was to explore how school leaders experienced their work during the COVID-19 school closure period in Estonia and Finland. The research was guided by the following question: Which aspects of leadership became vital in Estonian and Finnish school leaders’ work during the first remote learning period in 2020?

## Methods and materials

In April-May 2020, during the simultaneous remote learning periods in Estonia and Finland, researchers in both countries were separately preparing a nationwide data collection to gather information on how the unexpected changes had affected the daily practices of school leaders, personnel, students, and guardians. To gain a wider perspective of leadership, Estonian and Finnish researchers decided on a joint set of open-ended questions for school leaders to be integrated into their national surveys. In the context of this study, the concept of a school leader has been defined as a member of the school community who is involved in a formal leadership team. The data have been composed of school leaders’ responses to two open-ended questions: What was the most challenging for you as a leader in this situation [school closures]? What are the qualities required of a leader in a change like this? These questions were to complement each other and to trigger the thinking of the leaders and to reflect more broadly on the leadership in critical situations.

### Data sample and collection

In Estonia an electronic survey was sent to a sample of comprehensive schools (*N* = 166), representing all Estonian regions (*N* = 15), which is 31% of schools in Estonia. Altogether 219 school leaders from 138 schools responded to the survey. Most respondents (91%) worked at schools where the language of instruction is Estonian, whilst 9% reported Russian as the language of instruction. A third of the respondents (34%) worked at schools in the capital area, which demographically corresponds with the distribution of the Estonian population. The data were representative of the Estonian school leader population in terms of age (see Table 1) and the reported gender (78% female, 22% male). The professional experience of the Estonian participants varied from less than 5 years (24%) to over 20 years (41%).

**Table 1.** Age and work experience of the Estonian and Finnish participants.

Estonia (*N* = 219)										
Age (years)					Professional experience (years)					
−30	31–40	41–50	51–60	61–	1–5	6–10	11–15	16–20	21–	
3%	16%	32%	30%	17%	24%	14%	12%	9%	41%	
Finland (*N* = 775)										
Age (years)					Leadership experience (years)					
20–29	30–39	40–49	50–59	60–	0–5	6–10	11–15	16–20		21–
0%	7%	27%	54%	11%	21%	21%	22%	16%		20%

Background questions (e.g. work experience, age) were formulated differently or given different scales in the Estonian and Finnish surveys.

In Finland the electronic survey was sent to all comprehensive schools (*N* = 2231) in all Finnish municipalities (*N* = 309). The response rate was good (38%). The data are representative in the Finnish context covering 74% of the local education organizers (i.e. municipalities). Altogether 775 Finnish school leaders answered at least one of the open-ended questions and their responses were included in the study. Data collection was carried out in Finland’s two official languages, Finnish and Swedish, and 10% of school leaders answered in Swedish. The Finnish data are representative of the Finnish school leader population in terms of age (Table 1) and gender (56% female, 44% male). Leadership experience of Finnish participants was evenly distributed (Table 1).

### Data analysis

Two researchers in Finland and two in Estonia engaged in independent initial coding of the responses of the open questions to encounter issue-relevant meaning units, that is, small sections of text of one or several phrases long (Hsieh and Shannon, 2005). The meaning units were given sub-themes according to the main ideas they were carrying, for example, “I quickly organized a short training for my teachers to use online teaching platforms” was given the sub-theme “Technological encouragement.” Then the sub-themes, within a similar area, were combined under a broader main theme, for example, the sub-themes “Technological encouragement” and “Emotional support” formed the main theme “Giving support.” Subsequently, the researchers from both countries suggested a set of sub-themes which were then discussed. The formed sub-themes were compared to detect contradictions. Agreement concerning the coding was reached via multiple discussions during several rounds of analysis (Korstjens and Moser, 2018). The process yielded the creation of the Coding scheme (Table 2; for more detail, see Supplemental Annex) and resulted in identifying 13 sub-themes and 5 main themes.

**Table 2.** Coding scheme.

Main themes	Sub-themes
1. Making decisions	Structured leadership
	Sharing in leadership
	Risk management
2. Re-organizing schoolwork	Implementing new technology-related practices
	Re-organizing teaching practices
3. Communication	Internal communication
	External communication
4. Giving support	Technological encouragement
	Emotional support
5. Self	Self-management
	Managing stress
	Envisioning
	Trusting others

The credibility was increased by constant peer-checking and comparison, reliability of the main themes and sub-themes was decided by consensus (Creswell and Poth, 2018). One of the researchers is fluent in both Estonian and Finnish, which enabled cross-checking during the analysis process (Korstjens and Moser, 2018). Interrater reliability between two of the coders was calculated using Cohen kappa. The interrater reliability between Estonian coders was 0.780 (*p* < 0.001), 95% CI (0.664, 0.897), which is considered as “good” (Cohen, 1988). Also, the analysis of the Finnish data showed good interrater reliability with a value of 0.684 (*p* < 0.001), 95% CI (0.617, 0.751).

### Ethical considerations

Research participants in Estonia and Finland were provided with information about the key elements of the national studies, their rights as respondents, and the storage, analysis, and the use of the data. In Estonia the study went through the ethics committee of the Tallinn University to get approval for data collection. The researchers of the University of Helsinki are committed to following the Ethical Principles of Research in the Humanities and Social and Behavioural Sciences (TENK, 2021). A statement of the ethics of a research design must be requested from the University of Helsinki’s Ethical Review Board if a study meets any of the following: participants under the age of 15, exposure to exceptionally strong stimuli, a risk to cause mental harm or involving a threat to the safety of participants. This study did not include any of these items.

## Findings

The overview of the emphasis in responses to the open-ended question shows (Table 3) that many leaders shared their experiences related to themes of communication, giving support, and self. Next, we discuss the findings by reflecting on school leaders’ viewpoints drawn from their responses to the two questions. Under each theme, we first describe the elements related to it in the data, then we look at leaders’ viewpoints in more detail, per country.

**Table 3.** Percentages of the responses included in each of the coded themes.

		1. What was most challenging for you as a leader in this situation?		2. What are the qualities required of a leader in a change like this?	
		Estonia (%)	Finland (%)	Estonia (%)	Finland (%)
Making decisions	Structured leadership	1.4	0.8	28.4	4.8
	Sharing in leadership	1.1	0.7	4.2	2.8
	Risk management	0	1.7	6.2	4.1
Re-organizing schoolwork	Implementing new technology-related practices	11.8	1.8	9.0	3.2
	Re-organizing teaching	14.7	8.8	0.5	12.1
Communication	Internal communication	43.1	7.3	11.4	32.8
	External communication	1.9	14.1	4.7	11.5
Giving support	Technological encouragement	23.7	2.4	2.4	5.7
	Emotional support	13.7	11.9	10.0	49.0
Self	Self-management	11.8	24.6	2.4	50.7
	Managing stress	5.7	29.8	0.9	17.1
	Envisioning	1.9	1.6	0	5.5
	Trusting others	0	0.7	0.9	3.6

### Making decisions

Under this main theme we looked at the ways school leaders made decisions, the pace of making them, and the aspect of sharing in these processes (Table 3). The first of the three subthemes, *Structured leadership* was related to authoritarian style of organizing the schoolwork entailing giving clear and strict instructions to personnel. Second, *Sharing in leadership* practices concerned distribution of responsibilities and opportunities for joint discussions. In addition, some responses contained mentions of *Risk management* referring to situations in which leaders felt how they led the school through the crisis by making hasty decisions without enough knowledge or experience. Of the three subthemes, school leaders reported more structured than shared leadership or risk management, yet the emphasis varied in the responses per country.

The Estonian school leaders placed strong emphasis on structured leadership in their responses. They reported that the most important thing for the teaching community was to receive clear and strict instructions about what to do and how to act. Therefore, the decisions had to be contemplated, tangible, and clearly formulated. Sometimes the only thing “What is needed is the courage to decide, a bad decision is better than no decision at all” as one leader (EST48) stated. In the crisis, an authoritarian (or even autocratic) way of leading was sometimes perceived as necessary, especially if there was no time for a shared discussion with others. Some leaders considered essential to form a crisis management team having competence and power to make decisions and implement them. As a sub-theme, sharing in leadership was related to leaders’ feelings of not being able or willing to make all decisions by themselves, having trust in teachers, and encouraging teachers to solve problems on the spot.

The mentions of decision-making were relatively scarce in the Finnish school leaders’ responses if compared to the other main themes (Table 3). The leaders emphasized leader-centered (structured) leadership, which was often seen as an exception to the ordinary way of leading the school community: “Putting leader-led leadership in the front was something that does not happen in an expert organization on a daily basis, not even weekly” (FIN704). Further, leaders noted that there were less opportunities for a joint dialogue, and they had to decide on things without first discussing them with the teachers. Also, it seemed like the leaders had to supervise teachers’ work regarding the quality of distance teaching, and sometimes even in a strict manner to unify teaching practices. Few Finnish leaders wrote about sharing in leadership, yet some of them described how the situation called for more frequent meetings with the leadership team consisting of a leader and a group of teachers.

### Re-organizing schoolwork

The rapid shift to remote learning led to implementation of new practices, and many practices of schooling had to be re-organized. The Re-organizing schoolwork category covered two sub-themes of which *Implementing new technology-related practices* focused on the (new) technologies and platforms leaders introduced and implemented in their schools for teaching, learning, and other daily practices (e.g. meetings). The other sub-theme, *Re-organizing teaching* concerned leading the pedagogy needed with the new technologies and re-thinking the ways to organize a school day and re-allocating timetables. Overall, our observations indicate that the Estonian school leaders reflected on both the implementation of new practices in their schools and reorganization of teaching practices, whereas their Finnish colleagues focused on the reorganization of teaching practices (Table 3). Under this theme we also discovered that organizing the delivery of free school meals to students was part of the daily practices requiring re-scheduling in both countries.

In Estonia, school leaders considered it urgent to agree on the routines for schooling that all in the school community (i.e. all staff, students, guardians) could easily follow. They found it challenging to come by and implement new digital platforms and technological solutions, but also to share and store digital teaching materials to make them accessible to students. The introduction of platforms for meetings, finding ways for teachers to communicate with students, give them feedback and assess them were regarded as a hurdle. Moreover, several daily issues kept leaders busy, such as offering digital devices to underprivileged students, managing licenses and accounts, and organizing the admission of future students. In their responses the leaders considered the future and pointed out the need for clearly specified channels of communication and platforms for digital learning materials organized at the national level. In addition, the challenges they perceived that leading school life in an unknown situation included dealing with students who had lost their motivation, and practices enhancing students’ well-being (e.g. outdoor activities, online school events). They also pointed out that teachers who earlier worked autonomously in their classrooms, found it difficult to work individually and to give up old work habits. Further, leaders had to work closely with teachers in developing common principles for online assessment, student workload, and enriching teaching methods.

The Finnish leaders noted that to lead remote learning, a clear pedagogical vision, and an ability to apply it to the new situation were necessary. The concept of “pedagogical leadership” emerged often in the responses and referred to leaders’ response to teachers’ need for guidance regarding the structure of a school day and the requirements for student learning. One leader (FIN600) wrote that “In the beginning, the role of pedagogical leadership became more obvious as some teachers tended to demand too little from the students while others demanded too much.” Some leaders mentioned the importance of deciding on joint practices and the assessment of them, which enabled them to adjust the schoolwork according to the received feedback. In addition, some stated how it was essential that they were knowledgeable in the use of technology in schoolwork. They implemented new technologies for communication with teachers, among teachers, and between teachers and students. Some schools provided laptops or computers to students who did not have them at home.

### Communication

Under the theme of communication, we focused on descriptions about various forms of the guidelines, and interactions within the school, with colleagues in other schools, and educational administration and other stakeholders in the local community. The theme of communication with its two subthemes, *Internal* and *External Communication*, was an oft-repeated topic in both countries (see Table 3). The importance of clarity regarding the information and guidelines to teachers, and the interaction with families were highlighted relatively often by the leaders.

Among the Estonian respondents, Internal communication was perceived as the most challenging aspect of leading a school during the months of remote learning. It seemed to be demanding to facilitate and provide a shared (online) space for teacher collaboration. Although the organization of regular online meetings became a routine, finding ways to prevent teacher burnout caused by extensive online work was considered difficult. One leader (EST98) put this in words as follows:

Every morning at 10 we opened a virtual staff room with a less formal atmosphere. We had online meetings twice a week: on Tuesdays separate ones for teachers in lower and upper grades, and on Fridays a joint meeting for all. Once a week we had a meeting for the school management.

When describing communication with parents during the early phases of the pandemic, leaders emphasized launching of information channels and mutual communication. In addition, the formulation of guidelines for the members of the school community felt demanding while being surrounded by contradicting information coming from various sources.

In the Finnish context school leaders used expressions such as precision, transparency, and regularity when they described giving instructions and guidelines to teachers, students, and homes. In many responses the leaders underlined the requirement of clarity in the internal communication, and it was vital to possess “the ability to communicate in a clear and reassuring manner” (FIN585). It was the leader’s responsibility to filter out pieces of information that were not relevant for their school as there were various channels (including media) producing information and instructions. Although the aspect of providing guidelines was emphasized, internal communication was also about interaction and informal communication within the school community. Most challenges were related to the demand to keep contact with all members of the school personnel. External communication was often described as burdensome. The Finnish leaders reported struggling with the rapidly changing instructions coming from the authorities at state and local/municipal levels. These instructions were sometimes contradictory, and it was difficult to work out the best way to instruct the school community. Leaders expressed a need for centrally given instructions for all schools in the same local area. Some even wished for stronger leadership from the Ministry of Education. Furthermore, respondents suggested that local authorities should facilitate networking and collaboration between schools, which could help school leaders formulate shared guidelines and create local level coherency in school leadership.

### Giving support

The aspect of giving support emerged in the responses relatively often (Table 3). We noticed that the leader’s support to the school community was described in two ways, one of which was *Technological encouragement* and the other *Emotional support*. Technological encouragement covered students and the teachers, and it was about organizing short training sessions for the use of new technologies (e.g. teaching/learning applications, software), encouraging and providing informal guidance for using them. The emotional part of support involved listening to teachers, students, and guardians. The leaders felt a strong urge for being available, and to act in a sensitive manner and provide safety. Emotional support covered leaders’ practices aimed at creating a feeling of security among teachers and students. In general, emotional support was included in the responses more often than technological encouragement. Leaders experienced giving emotional support both as a challenge and as something that was expected from them at that moment, and it was reflected often, especially by the Finnish school leaders whereas the Estonian leaders focused more on technological encouragement.

According to the Estonian leaders, teachers needed technological encouragement in finding digital solutions and suitable platforms. The leaders noted that teachers afraid of online teaching needed guidance and support, and early career teachers seemed to require special attention. Regarding emotional support, the leaders reported how through offering constant attention, support, and by addressing teachers’ problems they tried to foster the feeling of community and the school spirit. One leader (EST12) wrote: “I tried to be supportive and mediate success stories and good experiences. I was there just to listen to teachers’ worries and comfort them. I told them to take care of themselves.” Leaders also considered it essential to support students’ sense of belonging to the school and had offered informal meeting spaces for students. Sometimes stressful situations at home arose, and the guardians needed reassurance. In some cases, the leaders sought professional help: “Through many interactions we tried to solve upcoming problems in a peaceful way. We involved a social worker and a psychologist to help these families” (EST62).

In the Finnish data, emotional support was one of the two themes emerging in the responses most often (Table 3). Leaders reflected on actions required to strengthen the feeling that they were available, they listened to and supported teachers. They described how they aimed to be sensitive to the changes in the atmosphere and how they “had to keep up the positive mood especially for the teachers having lack of trust on their capability to pull through the new situation” (FIN170). Concepts of wellbeing, empathy, encouragement, and patience were included in many responses. The leaders felt responsible for signaling stability in an unstable situation, which showed in responses like “It was important to convey an impression that despite the situation, the management is stable, and everything is going to be alright” (FIN685). Sometimes leaders were balancing between the expectations set for their role and their ability to meet these expectations. For example, keeping personal contact with every teacher and strengthening the sense of community were seen both as important and as challenging. Regarding technological encouragement, leaders focused on helping teachers, students, and homes in the use of technology in teaching and learning. Further, the ability to observe teachers’ ways of applying the new practices was essential because there were variations in teachers’ knowledge and skills regarding remote learning: “instructions and within school training and sharing were needed to level out the gap in teachers’ digital capabilities” (FIN169).

### Self

This theme was formed from four sub-themes reflecting leaders’ individual strategies for confronting and coping in the new situation (Table 3). School leaders referred to *Self-management* skills when they described the practice of prioritizing and organizing tasks into controllable units. They also wrote about being flexible and creative in regulating one’s work and time. Regarding the subtheme *Managing stress* leaders emphasized facing increasing workloads, lack of time, feelings of uncertainty, a sense of loneliness and continual changes caused by the constantly evolving situation. *Envisioning* has been composed of descriptions about predicting the near future, understanding the big picture, and acting proactively accordingly. *Trusting others* emerged in leaders’ expressions of the importance of being able to rely on their teaching community and teachers’ performance. Descriptions of Self-management and Managing stress were included in leaders’ answers relatively often, whereas Envisioning and Trusting others were seldom reported.

In the Estonian context the leaders considered *Self-management* to be an important aspect in coping with an unknown situation. They mentioned prioritizing tasks, drafting a clear plan for daily activities, and finding time for themselves and for their families. One respondent (EST101) described this as follows: “Combining my personal and professional life was hard. I had to implement a certain routine to be able to work and to spend some time with my children. I used to work in the evenings.” Regarding Stress management, the Estonian leaders reflected on the need to take care of their own health. Some leaders brought up parent encounters with negative overtones, which seemed to be psychologically difficult. Although they were supporting their school’s teaching community, leaders missed discussions with peers and often lacked support from municipal education administration: “When alone, you feel a growing need to discuss matters with an outsider, preferably with municipal administrators” (EST125).

Half of the responses in the Finnish data had a stance on *Self-management* as an essential quality required of a leader. The participants considered the ability to distinguish the crucial duties from the ones that could be postponed being important. Typical themes emerging in the responses included flexibility regarding working hours and tasks, adaptation to demands, agility to react, re-organizing own duties, and directing one’s own workload and wellbeing. Leaders working in small schools (less than 150 students) and having double roles as principals and teachers, reported the need to prioritize duties, as their workload had increased tremendously. One respondent described the situation as follows: “having a school with 100 students, over 10 teachers to supervise, my own class to teach, and duties of a principal—it required a lot of planning and preparation” (FIN234). Under this sub-theme, the challenges included features similar to the ones described as essential for pulling through the situation as a leader. Regarding Stress management, the leaders expressed their worries about their own wellbeing and that of teachers and students. This challenged the leaders in several ways, and many of them reported that they could not be certain “how teachers were doing their work, how they communicated with the students, how the students were coping with distance learning, and what had happened to the dropouts” (FIN150). Consequently, the Finnish leaders experienced feelings of inadequacy and sometimes loneliness. Trusting others and envisioning were scarce subthemes, yet we observed them more often in the Finnish responses than in the Estonian. Some leaders reflected on the importance of being able to understand the big picture and having wide knowledge of the educational field.

## Discussion

The Estonian and Finnish school leaders’ self-reported experiences stressed the themes of communication, giving support to the school community, and the leader’s ability to lead themselves and manage stress. These aspects signaled the leaders’ approaches as being composed of caring and empathy. We draw together and discuss the findings through four themes. We also bring out practical implications that would be helpful in developing leadership practices for leaders and teachers alike, in school communities in times of both turbulence and tranquility.

### Critical situations call for swift methods for decision-making

The process of making decisions is one of the foundations of leadership whether we focus on school leaders’ individual or collective leadership (e.g. leadership team) actions or leaders’ methods for ensuring and supporting teacher teams in their joint decision-making (Wang, 2021). Literature on crisis leadership underlines collaboration along with distribution of responsibilities and leadership tasks (e.g. Beauchamp et al., 2021; Harris and Jones, 2020). Nonetheless, regarding leaders’ work, this aspect did not explicitly emerge in our analysis. On the contrary, we discovered leader-centered practices which were more evident among the Estonian research participants. This approach, along with crisis management, enabled the rapid (and sometimes even hasty) pace in decision-making when continuously changing situations provided no time for shared discussions or collective processes. Consequently, the Estonian and Finnish leaders experienced time pressure and social isolation that may have contradicted their mindsets typically based on the importance of interaction and negotiation in decision-making (Eisenschmidt et al., 2021; Lahtero et al., 2019). Although the collective practices were not traceable in relation to explicit descriptions related to leading, they could be found elsewhere. Leaders reported methods for organizing various online platforms and channels enabling interaction and collaboration within the school community—including all teachers, other staff, and students.

Clear guidance coming from the leaders can be of utmost importance in critical situations creating the feeling of safety in an organization (Berger and Johnston, 2015). However, not all decisions can be leader-dependent—leaders should be able to trust teachers to take responsibility at the class-room level both individually and collectively. In general, leaders having a clear vision for their school and its development are able to guide collaboration in a systematic and diverse manner, which can result in well-functioning teams that can act responsively without waiting for leader’s instructions (Ahtiainen et al., 2021; Poom-Valickis et al., 2021). Consequently, the level of self-direction and sense of community of a school depends on the already existing ways of working and teachers’ earlier experiences. Presumably school communities, in which teacher collaboration, innovation, and efforts have been supported and encouraged, teachers are more likely to take the initiative (e.g. the leadership role) even in an unexpected situation (Eisenschmidt et al., 2021; Katzenmeyer and Moller, 2009).

Crises can provide schools with the potential to develop, yet it requires leaders to redirect their focus from minimizing damage and restoring order to innovating, giving up old habits and redirecting their practices (McLeod and Dulsky, 2021). Our findings indicate that the leaders could probably not employ the structures and practices that the collective decision making is usually based on in their schools. These structures did not necessarily function during the school closures. Consequently, leaders were forced to take on more responsibility. To tackle this challenge in the future, plans for school closures or structures for crisis leadership could be developed. One option is a creation of strategies for decision-making that involve teachers and enables a small group of teachers to participate in decision-making processes and take crisis leadership roles entailing pre-determined tasks (e.g. communication) ensuring the benefits commonly associated with distributed leadership (Harris, 2008; Hopkins, 2009; Spillane, 2005). That practice may allow the leaders to concentrate more on the big picture and necessary safety precautions. Anyway, to develop a school community in that direction, the decision-making processes should be reconceptualized and reflected through situations that exist in school contexts. That process could increase the understanding of the variety of occasions claiming attention for decisions targeted at the different layers in a school (e.g. classroom, school-home relations) and encourage teachers to solve problems in a timely manner.

### Pedagogical knowledge of a leader creates the ground for meaningful anticipation of coming (critical) situations

Turbulent times and crises are characterized by sudden turns in situations. They make future requirements for teaching and learning increasingly unpredictable, providing numerous options for the next steps, but often without a certainty of their correctness (Berger and Johnston, 2015; Dunn, 2020; Gurr and Drysdale, 2020). A rapid shift to new or modification of existing practices may force some level of learning for all, yet it can also push teachers too far out of their comfort zone.

School leaders participating in this study reported how they tried to tackle the challenges of distance learning by introducing new devices and technology-based practices. They were guiding teachers about new working methods. Both these channels require understanding of pedagogy and classroom work in re-organizing schooling in a meaningful way. The leaders’ descriptions of guidance for enrichment of online teaching, assessment methods, and requirements for student learning reflected a pedagogy-oriented approach to leading. This observation adds to the discourse around training and qualification requirements for school leaders and principals, and specifically focuses on the question of the meaning of studies in pedagogy or teacher qualification for the position of a leader in the context of education (Eisenschmidt et al., 2021).

Leaders in Estonia and Finland often describe teachers as autonomous actors at the classroom-level (Ahtiainen et al., 2021; Eisenschmidt et al., 2021). The school closures brought forward a new phenomenon as the leaders observed how teachers struggled in their everyday teaching work and needed support for pedagogy and assessment. Consequently, a determination of a leader in setting clear pedagogical guidelines for instruction and introduction of functional technological solutions was necessary. This determination reflects the argument of “technology as panacea ethos” accepted as the principle for implementation of new devices and applications, which in this case refers to seeing technology as the solution to all problems providing access to schools’ daily activities despite the closure of the school premises (Lindblad et al., 2021).

### Creating an emotionally stable environment is vital in unpredictable circumstances

In the processes of creating the feeling of safety or encouraging stronger involvement of teachers in decision-making, the emotional component is an integral part of leadership (Wang, 2021), and timely and well targeted internal communication is one tool for creating them (Marshall et al., 2020; Smith and Riley, 2012). In the current study the leaders described how they communicated to others that everything was under control and were urged to create stability and a feeling that there is a leader who is informed and knows the direction. It is also important to create virtual environments to support the creation of an emotionally inclusive environment. Thus, the communication was not limited to providing information and guidelines but extended for the purposes of showing sensitivity and empathy. Therefore, internal communication can become an essential channel for the school community to support each other (Beauchamp et al., 2021).

In a school community, the leader’s task is to focus on ensuring that stress is reduced for all members along with managing their own emotions and wellbeing (Mahfouz, 2020). In the current study, leaders found themselves responsible for the wellbeing of students and teachers. These aspects resonate with one of Reid’s (2021) findings suggesting that leaders’ personal beliefs about how to best manage their own stress are aligned with the idea that they should work relentlessly to support all staff within their organization. Moreover, in one of our earlier studies we found that virtues seem to guide leaders to move towards desired goals, supporting others, and solve challenging situations in a morally sustainable way (Eisenschmidt et al., 2019). During the pandemic, new practices were experienced and tried out, and some of these may continue and become an everyday leadership practice. Therefore, we should follow-up whether some of the emotional components or new ways of working will keep developing in the schools. Furthermore, the established communication and collaboration platforms could widen teachers’ opportunities for meetings or even observation of each other’s lessons through conference calls in the future. While we can see promising opportunities in these digitalized forms of working, we must be cautious about their implementation and development and further discuss the purposes they would be serving in the field of education and the other parties surrounding it (e.g. corporate, and commercial actors; Cone et al., 2022).

### Peer-support and networks are essential for leader’s work

School leaders are public figures in their local communities and their actions are explicit to all school community members. They are often expected to take full responsibility for solving conflicts and to fulfill all their duties with considerable urgency, which can be emotionally exhausting (Mahfouz, 2020). The personal characteristics of a leader, like resilience, self-management, and stress-management, become important in situations departing from the usual ways of schooling (Smith and Riley, 2012; Southwick et al., 2017). Leaders have experienced increased levels of stress and anxiety since the early days of COVID-19 (Reid, 2021). To put on a brave face for the members of the school and local community, leaders have controlled or suppressed their own stress and anxiety (Reid, 2021). Further, in the current study, the management of everyday duties and flexibility were found necessary, but also challenging. The importance of managing workload and the emotional burden related to the uncertain situation were emphasized especially in the responses of Finnish leaders. Although leaders may have a stronger social and moral responsibility for taking care of others, they would need to prioritize their own wellbeing and look after themselves (Southwick et al., 2017).

Networks and other structures enabling encounters with leaders from other schools are important in general. These professional channels can become essential in times of crisis, and enable a space for sharing worries, and getting support from peers facing similar challenges (Reid, 2021). Our study made visible leaders’ feelings of loneliness and need for collaboration networks and support outside their schools. Although the main guidelines during the school closures were given by the state and health authorities, a relatively lot of autonomy regarding their realization was placed at the level of local education administration (e.g. municipalities) and schools (Hargreaves and Fullan, 2020; Lindblad et al., 2021). Therefore, the actions taken by the local education administration were more focused on day-to-day survival, and in some areas, that overlooked individual leaders’ needs for professional and emotional support. One may argue that in municipalities which already had established collaboration networks and other structures for sharing before the COVID-19 pandemic, leaders were supported and avoided the strong feelings of professional isolation.

As the lack of local level collaboration and support appeared to be a factor challenging the leaders, more focus could be put on facilitating the interaction between the schools. If planned carefully, the structures for horizontal collaboration can function as places for distributing crucial information and provide peer support. Environments like these that may not only offer resources for coping with the critical situations but also create coherence in practices across schools locally and through that enhance educational equality. Moreover, lessons from the COVID-19 pandemic period regarding support to teachers, pedagogical innovations, and novel ways for teaching could be spread through collaboration between school leaders. Being proactive in preparing for future challenges entails intentionally looking for and discussing the ways others have reacted during the times of crisis and its aftermath.

### Future research

There are at least two directions for further research. First, we need follow-up studies to understand how the continuation of the pandemic has developed leadership practices, which of the developments had emerged at the beginning, and which are more entrenched in school culture. Secondly, the teachers’ perspective would deepen the understanding of leadership practices regarding leaders’ coping, support, and organizational climate.

## Limitations

Although the researchers were careful in the formulation of the two open-ended questions in their national languages, the questions were placed in a different order in the national surveys. The Estonian researchers asked first about challenges (What was most challenging for you as a leader in this situation) and the Finnish about the leadership qualities (What are the qualities required of a leader in a change like this). After noticing this slip-up, this was considered when interpreting the results, yet it may have led to a certain emphasis in the responses. Nonetheless, the questions were adjacent in both surveys, aiming to complement each other and helping leaders to reflect more broadly their work.

## Supplemental Material

sj-docx-1-eer-10.1177_14749041221138989 – Supplemental material for Leading schools during the COVID-19 school closures in Estonia and Finland Click here for additional data file. Supplemental material, sj-docx-1-eer-10.1177_14749041221138989 for Leading schools during the COVID-19 school closures in Estonia and Finland by Raisa Ahtiainen, Eve Eisenschmidt, Lauri Heikonen and Merilyn Meristo in European Educational Research Journal
