# Supplementary material for: Leading schools during the COVID-19 school closures in Estonia and Finland
Source: Eur Educ Res J. 2022 Dec 2:14749041221138989. doi: 10.1177/14749041221138989 (PMC9720469; doi:10.1177/14749041221138989)
Supplement: sj-docx-1-eer-10.1177_14749041221138989 – Supplemental material for Leading schools during the COVID-19 school closures in Estonia and Finland [file sj-docx-1-eer-10.1177_14749041221138989.docx]

Annex. Coding scheme

|  | | |
| --- | --- | --- |
| Main themes | Sub-themes | Description |
| 1. Making decisions | Structured leadership | Giving direct instructions; strict or authoritarian style of instructing teachers and other staff members |
|  | Sharing in leadership | Sharing responsibilities and roles in decision-making; organising joint discussions for decision-making |
|  | Risk management | Managing the critical situations, leading the school through the crisis; making quick decisions without enough knowledge or previous experience |
| 2. Re-organizing school work | Implementing new technology-related practices | Implementing and organizing technology-related platforms for meetings with teachers and for other types of interaction |
|  | Re-organizing teaching practices | Implementing practices needed for the use of technology in teaching and learning; re-allocating timetables; re-conceptualizing daily practices in schools |
| 3. Communication | Internal communication | Providing guidelines for schooling; forms and quality of communication; interaction with teachers and homes |
|  | External communication | Guidelines coming from the ministry, local educational administration, or other authorities; meetings and communication with local education administrators or other principals and schools in the area |
| 4. Giving support | Technological encouragement | Encouraging teachers and students in the use of new technologies; organizing short, quick trainings for teachers (e.g., learning/teaching apps, software) |
|  | Emotional support | Supporting and encouraging emotionally teachers and students; being available and listening to people; providing a sense of security |
| 5. SELF | Self-management | Prioritizing tasks, organizing them to a manageable form, linking overlapping tasks; steering own workload; adaptability |
|  | Managing stress | Acknowledging lack of time, increased workload; need for support from colleagues and superiors; feelings of uncertainty and isolation |
|  | Envisioning | Trying to predict happenings and acting accordingly |
|  | Trusting others | Trusting teacher community, their performance and willingness |
